# Chinese Giant Salamander Iridovirus 025L Is a Viral Essential Gene

**DOI:** 10.3390/v15030617

**Published:** 2023-02-23

**Authors:** Zijing Liu, Daofa Xie, Shirong Nong, Yingzi Wu, Suxian Huang, Xianhui He, Tianhong Zhou, Wei Li

**Affiliations:** 1 College of Life Science and Technology, Jinan University, Guangzhou 510642, China; 2 Southern Marine Science and Engineering Guangdong Laboratory (Zhuhai), Guangzhou 510632, China

**Keywords:** ranavirus, essential gene, transcription, replication, deletion mutation

## Abstract

Ranavirus is a large nucleocytoplasmic DNA virus. Chinese giant salamander iridovirus (CGSIV) belongs to the ranavirus genus, and its replication involves a series of essential viral genes. Viral *PCNA* is a gene closely associated with viral replication. CGSIV−025L also encodes PCNA−like genes. We have described the function of CGSIV−025L in virus replication. The promoter of CGSIV−025L is activated during viral infection, and it is an early (E) gene that can be effectively transcribed after viral infection. CGSIV−025L overexpression promoted viral replication and viral DNA replication. siRNA interfered with CGSIV−025L expression and attenuated viral replication and viral DNA replication. The Δ025L−CGSIV strain with the deletion of CGSIV−025L could not replicate normally and could be rescued by the replenishment of 025L. CGSIV−025L was proven to be an essential gene for CGSIV by overexpression, interference, and deletion mutation experiments. CGSIV−025L was found to interact with CGSIV−062L by yeast two−hybrid, CoIP, and GST pulldown. Thus, the current study demonstrated that CGSIV−025L is an essential gene of CGSIV, which may be involved in viral infection by participating in viral DNA replication and interacting with replication−related proteins.

## 1. Introduction

Ranaviruses (family Iridoviridae, Alphairidovirinae, genus Ranavirus) are large, double−stranded DNA viruses that infect a variety of ectothermic vertebrates, such as amphibians, reptiles, and fish [1,2,3,4]. The Chinese giant salamander is the largest amphibian in the world and has great ecological and commercial value. Unfortunately, most giant salamander farms have experienced epidemics with high mortality rates since 2010, caused by giant salamander ranavirus and giant salamander iridovirus [5,6,7,8]. Chinese giant salamander iridovirus (CGSIV) is a new virus related to high mortality in giant salamanders. This virus was isolated from a diseased giant salamander at a giant salamander farm in Zhangjiajie, Hunan Province [8]. However, the mechanism by which viral genes affect viral replication has not been revealed. Several genes that may enhance viral replication were identified based on our previous studies using whole−genome sequencing, CGSIV analysis (accession number: KF51220), and a homology comparison with known databases [9]. One of these genes, PCNA, is considered to be closely related to the viral replication mechanism [8,9].

Proliferating cell nuclear antigen (PCNA) was first discovered by Miyachi in 1978 in the serum of patients with systemic lupus erythematosus [10,11]. Subsequent studies demonstrated that PCNA is closely related to cellular DNA synthesis. PCNA acts as a clipping loop to trap DNA strands and as a platform to recruit other protein factors to regulate DNA replication, and it also has other biological functions [12,13,14,15]. Viral PCNA is a spatially conserved protein, and such genes have been found in many double−stranded DNA viruses [16,17,18]. Moreover, viral PCNA also acts as a polymerase switch and recruitment factor that interacts with a variety of viral and cellular proteins to promote viral DNA replication [19,20,21,22]. PCNA is the core gene of iridoviruses [23], and there are open reading frames encoding PCNA in many ranavirus genomes that have been fully sequenced, such as Tiger frog virus (TFV) [24], Ambystoma tigrinum virus (ATV) [25], and Grouper iridovirus (GIV) [26,27,28,29]. Current studies on the expression, isolation, and structural functions of iridovirus PCNA include red sea bream iridovirus and rana grylio virus (RGV). PCNA was identified as a virion protein in RGV and specifically colocalized with the viral nucleic acid at the viral assembly site in the cytoplasm of infected hosts [30].

A previous study showed that PCNA−like proteins are detectable in CGSIV virion proteins by mass spectrometry [8]. The CGSIV PCNA protein is encoded by CGSIV−025L and contains 245 amino acids. However, the function of CGSIV−025L and its encoded proteins in viral replication remains unclear. At present, only CGSIV MCP [8], ATV 11R, and 53R [31] have been proven to be essential genes in ranavirus. Therefore, this study further investigated the molecular function of CGSIV−025L in the viral infection and replication of the cultured epithelioma papulosum cyprini (EPC) model in vitro. We investigated the effect of CGSIV−025L on viral replication by overexpression, siRNA knockdown, and a defective recombinant virus and screened the viral proteins interacting with CGSIV−025L. The results demonstrated that CGSIV−025L is an essential CGSIV gene, which may be involved in viral infection by participating in viral DNA replication and interacting with related proteins. This study will be helpful in elucidating the mechanisms of ranavirus infection and replication.

## 2. Materials and Methods

### 2.1. Viruses and Cells

CGSIV (Chinese giant salamander iridovirus) that was maintained in our lab were used in the present study [8]. The epithelioma papulosum cyprini (EPC) line was cultured in M199 (BI, Akko, Israel) medium supplemented with 10% fetal bovine serum (FBS, Gibco, Melbourne, Australia) at 26 °C until use [32]. To prepare virus stocks, EPC cells were infected with CGSIV at an MOI of 1. Three days postinfection, the infected cells were collected, freeze–thawed, aliquoted, and kept at −80 °C until use.

### 2.2. Plasmid Construction

Recombinant plasmids were constructed as described previously [32]. The corresponding primers were used to amplify different types of nucleic acid fragments (Table 1). Plasmids for overexpression, yeast two−hybrid (Y2H), coimmunoprecipitation (CoIP), and GST pulldown assays were constructed. All plasmids used in this study were verified and confirmed using DNA sequencing. Specific operations were as follows.

In order to construct a plasmid overexpressing CGSIV−025L, the coding gene of CGSIV−025L was amplified with primer 025L−F1/R1 and cloned into pEGFP−N1 to obtain the overexpression plasmid pEGFP−025L.

To construct bait plasmids for the screening of CGSIV−025L interacting proteins, the coding gene of CGSIV−025L was amplified with primer 025L−F2/R2 and cloned into pGBKT7 to obtain bait plasmids pGBKT7−025L. CGSIV−062L was amplified with primer 62L−F1/R1−HA and cloned into the pGADT7 plasmid to obtain pGADT7−062L.

CGSIV−025L was amplified with primer 025L−F2/R2, cloned into pcDNA3.1 (+), and constructed as pcDNA3.1−025L−Flag to generate plasmids for CoIP. CGSIV−062L was amplified with primer 62L−F2/R2−HA and cloned into pcDNA3.1 (+) to construct pcDNA3.1−062L−HA.

CGSIV−062L was amplified with 62L−F1/R1 and cloned into the pEGX4T7 plasmid to construct a prokaryotic plasmid pEGX4T7−062L expressing GST−062L protein.

### 2.3. Dual−Luciferase Reporter Assays, Drug Inhibition Assay

To investigate whether CGSIV−025L gene promoter transcription could initiate transcription, EPC cells were transfected with pGL3−All (−400/+34) recombinant plasmid containing the 025L gene promoter sequence using KpnⅠ and HindⅢ treatment. Using the transfected pGL3−basic plasmid as a control, after infection with the virus, Dual−Glo® Luciferase Reagent samples were prepared for detection, and a multifunctional microplate detector was used for the detection of experimental values. The sequences of primers 025L−F3 and 025L−R3 are shown in Table 1.

A drug inhibition test was used to detect the transcription phase. Cycloheximide (CHX), an inhibitor of de novo protein synthesis, and arabinonucleoside cytosine (AraC), an inhibitor of viral DNA replication, were used as suppressive agents during viral infections. Reverse transcription polymerase chain reaction (RT−PCR) analysis was performed with primers CGSIV−025L−F2/R2. The internal control gene β−actin and the known immediate early gene ICP−46 and late gene MCP were also analyzed using RT−PCR [23,33]. The specific primers of β−actin and the 025L, MCP, and ICP−46 genes are shown in Table 1.

The transcription phase of CGSIV−025L was detected using RT−PCR. EPC cells were infected with CGSIV multiple infection (MOI) at 1 or simulated infection, and EPC cells were collected at specified times after infection. RNA was extracted using TRIzol reagent (Life Technologies, Carlsbad, CA, USA) according to the manufacturer’s instructions. RNA was reverse−transcribed into cDNA using the Access RT−PCR System Kit (Promega, Madison, WI, USA).

### 2.4. Knock down CGSIV−025L

EPC cells were transfected with CGSIV−025L siRNA and NC siRNA (GenePharma, Shanghai, China) using PolyHigh Transfection (Sangon Biotech, Shanghai, China). Cells were infected with CGSIV and harvested at the indicated time points for RNA or DNA extraction. The targeted siRNA sequence of CGSIV−025L mRNA was as follows: (5′−GGGCAGUACACGAUGAAGATT−3′). The sequence targeted by the control RNAi plasmid was (5′−UUGGAAGCGACACGAGAGATT−3′).

### 2.5. Quantitative Real−Time PCR (qPCR) Detection

To investigate the effect of the upregulation and downregulation of the 025L gene on viral replication, EPC cells were transfected with overexpression plasmids and siRNA by PolyHigh Transfection in 6−well plates and infected with CGSIV. The cultures were collected 24 h after viral infection and viral RNA was extracted. qPCR was used to evaluate the transcription level of CGSIV−025L. The primers of CGSIV−025L are listed in Table 1. The internal reference genes were 18 s or β−actin. The expression of target genes was normalized to the reference genes and calculated using the 2^−ΔΔCt^ method. Reaction procedure: 95 °C, 30 s; 95 °C, 10 s; 60 °C for 34 s; 40 cycles.

To determine the viral genomic DNA copy number, viral growth was stopped by freezing cultures at the indicated time (16 h for overexpression and siRNA studies). The virus was released by three cycles of freeze–thaw, and the cell debris was removed by low−speed centrifugation. Viral genomes were extracted according to the Viral Genome Extraction Kit (OMEGA). CGSIV MCP gene primers were designed to evaluate CGSIV DNA replication [1], and the pMD18T−MCP plasmid (constructed and preserved in this experiment) was used as the standard. CGSIV MCP−F and CGSIV MCP−R were used as upstream and downstream primers, and the absolute quantitative PCR was performed on an ABI 7300 fluorescence qPCR instrument using Takara SYBR^®^ Premix Ex Taq™ II. The same steps were followed for the reaction procedure.

### 2.6. CGSIV Viral Titer

To determine the viral titer, viral growth was stopped by freezing the cultures at the indicated times (48 h.p.i. for siRNA studies and overexpression). Virions were released by three cycles of freeze–thaw and cellular debris removed by low−speed centrifugation. Clarified, virus−containing supernatants were serially diluted 10−fold and 200 μL of each dilution was added to duplicate wells of confluent EPC monolayers grown on 96−well plates.

### 2.7. Defective Virus Was Constructed by Homologous Recombination

The Puro−RFP fusion protein expression box was formed by connecting the purinomycin resistance gene with RFP via p2A. The expression box was used to replace the target gene PCNA and expressed under the regulation of the MCP strong promoter. puro−F/R, RFP−F/R, and PCNA−F3/R3 were used as primers to construct pGL3−MCPprom−ΔPCNA recombinant plasmids by bypass PCR and KpnI and HindIII treatment. Using puro−F/R and RFP−F/R as primers, the pcDNA3.1−Puro−p2A−RFP recombinant plasmid was constructed by EcoRI and XbaI as a positive control. The primer sequence is shown in Table 1.

Defective CGSIV was constructed by a method similar to that used to produce the recombinant Bohle iridovirus [34] and Ambystoma tigrinum virus [35]. CGSIV was repeatedly infected into cells transfected with recombinant plasmid pGL3−MCPprom−Δ025L until a single fluorescent plaque appeared. The fluorescent venom was collected, stored at −80 °C, and frozen and thawed three times. Purinomycin was used for multiple rounds of screening and purification. The recombinant plasmid pcDNA3.1−025L−Flag constructed above was used for the remediation experiment of the defective virus. Then, 24 h after the transfection of the pcDNA3.1−025L−Flag plasmid, the above hybrid virus containing red fluorescence was reinfected.

### 2.8. Interaction Analysis

Yeast two−hybrid experiment. The pGBKT7−025L bait plasmid and CGSIV genomic library plasmid PEG/LiAc were co-transformed into yeast strain AH109 to obtain a positive clone. The β−galactosidase activity and the autoactivation activity of bait protein BD−025L were analyzed, and then the proteins interacting with CGSIV−025L were screened out.

Coimmunoprecipitation: The eukaryotic inducible expression protein plasmid was constructed and transfected into EPC cells, and the cells were lysed with RIPA lysate for CoIP and Western blotting analysis, as previously described [36].

GST pulldown experiment. GST−062L bound glutathione agarose beads were incubated for 3 h with Hela cell lysates transiently expressing the pcDNA3.1−025L−Flag plasmid. It was first washed three times with RIPA lysis buffer, and then mixed with an equal volume of 2 × SDS loading buffer and boiled for 10 min. Immunoblot analysis was performed as previously described [37].

### 2.9. Statistical Analysis

All experiments in this study were performed in triplicate independently, and the data were expressed as mean ± standard deviation (mean ± SD). GraphPad Prism 7 software was used for statistical analysis of the experimental data, and Student’s t test (two groups) and one−way ANOVA followed by the Bonferroni post hoc test (multigroup comparison) were used for comparisons between groups. *p* < 0.05 indicates that the difference between groups is statistically significant, * *p* < 0.05, ** *p* < 0.01, *** *p* < 0.001; ns indicates no significant difference.

## 3. Results

### 3.1. Initiation Transcription and Transcriptional Expression of CGSIV−025L Gene

**Figure 1:**
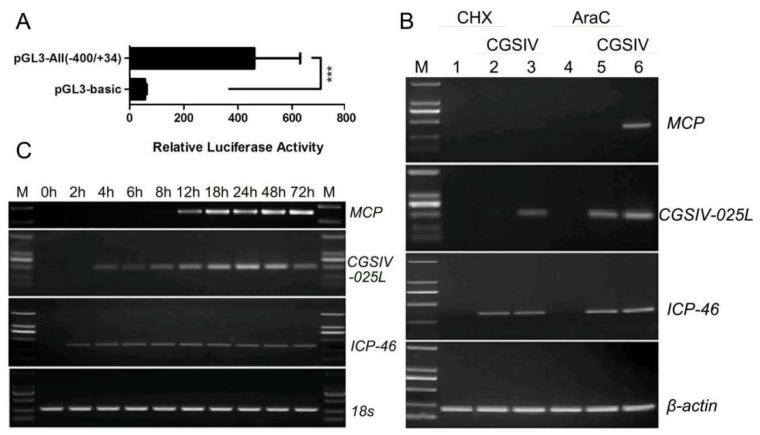
Transcription analysis of CGSIV−025L. (**A**) The promoter of CGSIV−025L was infected by virus to initiate transcription. (**B**) Determination of the transcriptional phase of CGSIV−025L under drug treatment. (**C**) Transcription of CGSIV−025L was verified using RT−PCR. RNA was isolated at different time points (0, 2, 4, 6, 8, 12, 18, 24, 48, and 72 h.p.i.) after CGSIV−HN11 infection, and transcription of CGSIV−025L was detected using RT−PCR. M is a DNA molecular weight marker, MCP is a late gene marker, and ICP−46 gene is a very early gene marker; 18S rRNA and β−actin gene were used as internal references. Data were expressed as mean ± SD (*n* = 3; *** *p* < 0.001).

To determine whether CGSIV−025L promoter pGL3−All (−400/+34) could initiate transcription, we examined the promoter activity of pGL3−All (−400/+34) and the empty vector pGL3−basic without the inserted promoter fragment after virus infection by dual−luciferase reporter assays. Dual−luciferase reporter assays showed that the CGSIV−025L promoter pGL3−All (−400/+34) exhibited stronger luciferase activity compared with the empty vector pGL3−basic without the promoter fragment after virus infection (Figure 1A), which was eight times higher than that of the negative control (*p* = 0.009, *p* < 0.01). The results showed that the promoter of the CGSIV−025L gene could initiate transcription during viral infection.

To determine the transcription phase of the CGSIV−025L gene, CHX and AraC were used for a drug inhibition assay. RT−PCR results showed that no transcription of the 025L gene was detected in the group without CGSIV infection (lane 1) or in the group with CHX inhibition after CGSIV infection (lane 2) (Figure 1B). At the same time, the transcription of the 025L gene was observed in the CGSIV infection group (lanes 3 and 6) and in the AraC inhibition group after CGSIV infection (lane 5), and the specific band of the reference control β−actin gene was present in all six samples. The results indicated that CGSIV−025L transcription was inhibited by the inhibitor of de novo protein synthesis, CHX, but not by the inhibitor of DNA replication, AraC. These results showed that the transcription phase of CGSIV−025L was an early (E) gene. The ICP−46 and MCP genes were universal immediate early and late gene controls for ranavirus, showing the IE and L gene transcription phases, respectively, as expected.

Seeking to investigate the transcription pattern of CGSIV−025L at different time points after CGSIV infection in EPC, RT−PCR analysis of time point expression showed that the CGSIV−025L transcript was identified as early as 4 h.p.i. (474 bp), and high levels of transcript expression persisted until 72 h.p.i. (Figure 1C). As a temporal control, the transcription of the very early gene ICP−46 was detected at 2 h, and the transcription of the late gene MCP was observed at 12 h. The 18S rRNA specific amplification band as a reference was present at all time points, and the expression level was essentially consistent. The results were consistent with the drug inhibition analysis, which confirmed that CGSIV−025L was an early viral gene and was transcribed.

In conclusion, the CGSIV−025L promoter can initiate transcription after viral infection, and CGSIV−025L starts transcription early in viral infection and continues to be highly expressed during viral infection.

### 3.2. Overexpression of CGSIV−025L Promoted Virus Proliferation

**Figure 2:**
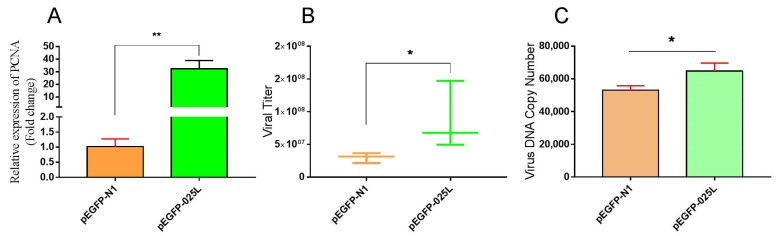
Effects of CGSIV−025L overexpression on viral and DNA replication. EPC cells were transfected with pEGFP−025L plasmid and pEGFP−025L plasmid, respectively, and cells were lysed 48 h after transfection. (**A**) Cell RNA was extracted and qPCR was used to determine the transcription level of 025L gene after reverse transcription; (**B**) virions were collected repeatedly by freezing and thawing, and viral titers were detected by TCID50 method; (**C**) the viral genome was extracted and the copy number of the viral genome was detected by absolute quantitative PCR. Data were expressed as mean ± SD (*n* = 3; * *p* < 0.05, ** *p* < 0.01).

CGSIV−025L was cloned from the CGSIV genome into the pEGFP plasmid. To determine the effect of plasmid (pEGFP−025L) overexpression, we extracted cell RNA after overexpression and performed qPCR after reversal. The results in Figure 2A show that the relative transcript level of the overexpressing CGSIV−025L group was approximately 43 times higher than that of the negative control group.

To investigate the effect of CGSIV−025L overexpression on viral replication, we collected the viral cell mixture after the overexpression of the pEGFP−025L plasmid, and we measured the viral titer via the TCID50 method. The viral titer assay, shown in Figure 2B, revealed that the average viral titer of the overexpressing CGSIV−025L group was 8.02 × 10^7^ TCID50/mL, and the average viral titer of the negative control group was 3.00 × 10^7^ TCID50/mL. The average viral titer of the overexpressing CGSIV−025L group was significantly higher than that in the negative control group. The average viral titer of the two groups showed a statistically significant difference in the T test (*p* = 0.044, *p* < 0.05). These data indicate that CGSIV−025L overexpression enhanced CGSIV viral replication in vitro.

Viral DNA replication is an important process in viral replication. The PCNA protein encoded by CGSIV−025L is closely related to viral DNA replication. To investigate whether CGSIV−025L overexpression affects viral DNA replication, after 16 h of overexpression of CGSIV−025L, we collected the viral cell mixture and extracted the viral genomic DNA. MCP was used as the standard, and the copy number of the viral genomic DNA was determined by qPCR. The results of viral DNA copy number measurement, shown in Figure 2C, indicated that the average viral genomic DNA copy number of the CGSIV−025L overexpression group was 6.47 × 10^4^, and the average viral genomic DNA copy number of the negative control group was 5.31 × 10^4^. The average copy number of viral genomic DNA in the CGSIV−025L overexpression group was significantly higher than that in the negative control group, and there was a statistically significant difference in the average copy number between the two groups in the T test (*p* = 0.023, *p* < 0.05). These data indicate that CGSIV−025L overexpression promoted CGSIV DNA replication. Therefore, we hypothesized that CGSIV−025L may promote viral replication by promoting viral DNA replication.

### 3.3. Silencing CGSIV−025L Attenuated Virus Proliferation

**Figure 3:**
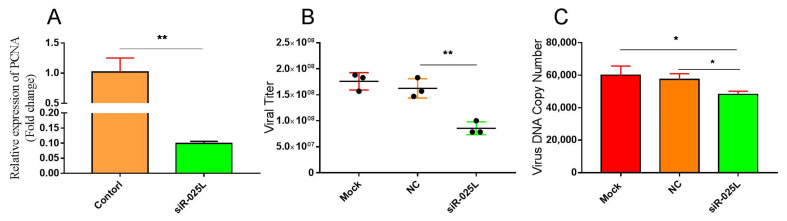
Effect of siRNA interference with CGSIV−025L expression on viral replication. CGSIV−025L targeting siRNA and NC were transfected into EPC cells, respectively, and cells were lysed 48 h after transfection. (**A**) RNA was extracted from cells, and the transcription level of CGSIV−025L was determined by qPCR after reverse transcription. (**B**) Virions were collected repeatedly by freezing and thawing, and viral titers were studied using the TCID50 method; (**C**) the viral genome was extracted and the copy number of the viral genome was calculated by absolute quantitative PCR. Data were expressed as mean ± SD (*n* = 3; * *p* < 0.05, ** *p* < 0.01).

To determine the knockdown effect of CGSIV−025L siRNA, cell RNA was extracted after the transfection of siRNA interfering with CGSIV−025L, and the transcription level of CGSIV−025L was examined by qPCR after reversal. The qPCR results shown in Figure 3A indicate that the transcription level of CGSIV−025L in the siRNA interference group was 10% of that in the negative control group. These date indicate that the siRNA could effectively knock down the transcription of CGSIV−025L.

To study the effect of CGSIV−025L gene knockdown on viral replication, we extracted cells after the transfection of siRNA interfering with the 025L gene and collected the viral cell mixture, and we measured the viral titer using the TCID50 method. The viral titer test, shown in Figure 3B, found that the average viral titer in the siR−025L group was 8.58 × 10^7^ TCID50/mL. The average viral titers of the NC group and blank control group were 1.62 × 10^8^ TCID50/mL and 1.76 × 10^8^ TCID50/mL, respectively. The average viral titer of the siR−025L group was 47% and 51% lower than that of the NC group and blank control group, respectively, which was significantly lower than that of the NC group. The viral titer of the siR−025L group was statistically significantly different from that of the NC group and blank control group in the T test (*p* = 0.004, *p* < 0.01). These results indicate that the knockdown of CGSIV−025L expression by siRNA attenuated the viral replication of CGSIV in vitro and the production of infected progeny CGSIV virions.

As mentioned above, viral DNA replication is an important step in viral replication. To investigate whether CGSIV−025L gene knockdown affects CGSIV DNA replication, after transfection of the siRNA interfering with CGSIV−025L, we collected the viral cell mixture, extracted viral genomic DNA, and measured the copy number of the viral genomic DNA by qPCR, using MCP as the standard. The results of qPCR, shown in Figure 3C, indicated that the average viral genomic DNA copy number of the siR−025L group was 4.82 × 10^4^, and the average viral genomic DNA copy number of the NC transfection group and blank control group was 5.74 × 10^4^ and 6.00 × 10^4^, respectively. The average viral genomic DNA copy number of the siR−025L group was reduced by 20%, and it was significantly lower than that of the NC group and blank control group. The T test showed that the copy number of the siR−025L group was significantly different from that of the NC group (*p* = 0.016, *p* < 0.05). These results showed that the knockdown of CGSIV−025L expression by siRNA attenuated CGSIV replication and viral DNA replication.

### 3.4. Deletion of CGSIV−025L Inhibited Virus Proliferation

**Figure 4:**
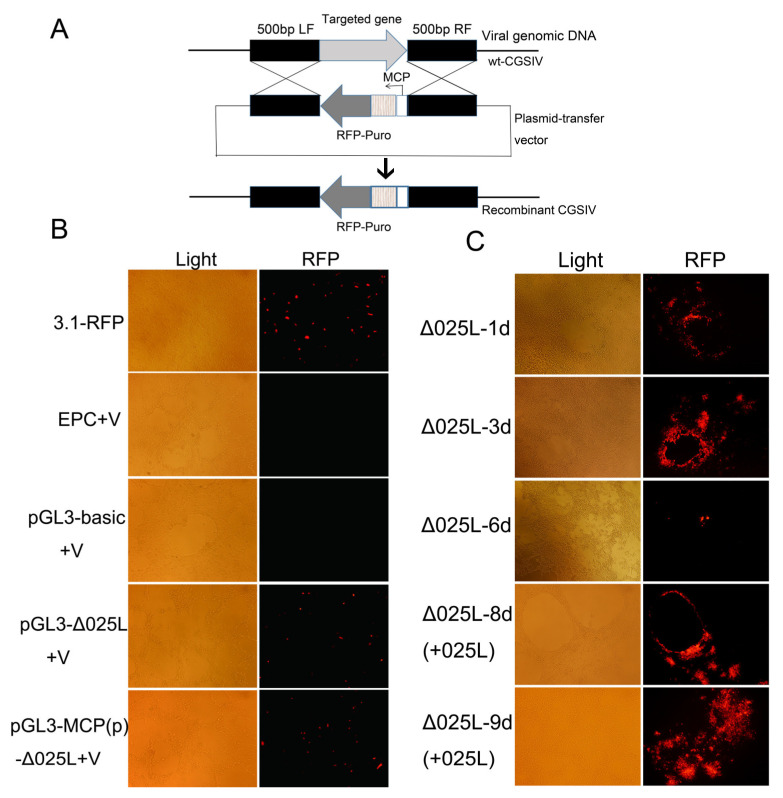
Construction, detection, and screening of Δ025L−CGSIV recombinant defective virus strain. (**A**) The cassette consists of a fluorescent reporter gene, RFP, fused to a resistance gene, puro via a short linker, controlled by ranavirus or the ectopic promoter MCP. The cassette is flanked by the left and right sequence portions (500 bp) of the target site and cloned into an Escherichia coli plasmid. Cells were transfected with liposomes and then infected with wild−type herpes virus to produce homologous recombination. In the presence of the drug, selection was sequentially performed by viral replication, followed by the isolation of fluorescent plaques. (**B**) Detection of red fluorescent protein expression in cells. (**C**) Screening and reverting mutation of Δ025L−CGSIV recombinant defective virus strain. The letter “d” represents the number of proliferating generations in defective virus screening.

Both the overexpression and knockdown of CGSIV−025L had important effects on viral replication, indicating that the expression level of CGSIV−025L in viral replication may affect viral replication by affecting viral DNA replication. To further study the effect of CGSIV−025L deletion on virus replication, a defective virus (Δ025L−CGSIV) was constructed by homologous recombination technology, and the technical diagram is shown in Figure 4A. The result of the overexpression of the recombinant plasmid, shown in Figure 4B, indicated that pcDNA3.1−Puro−p2A−RFP in the positive control group showed fluorescence, which confirmed that foreign gene RFP could be expressed in EPC cells. No red fluorescence was observed in blank control EPC + V cells and negative control pGL3−Basic + V cells. The overexpression of recombinant plasmid pGL3−Δ025L showed no fluorescence, but, after infection, fluorescence appeared. The recombinant plasmid pGL3−MCP (p)−Δ025L overexpressing the MCP strong promoter showed red fluorescence after virus infection, and the expression of red fluorescence was greater than that of the 025L promoter group.

To screen Δ025L−CGSIV and study the effect of the defective virus on virus replication, we screened CGSIV−025L defective virus strains by transfecting the MCP promoter with recombinant plasmid MCPprom−puro−p2A−RFP. Homologous recombination screening, shown in Figure 4C for Δ025L−1d, indicated that the cells of the first generation of the defective recombinant virus showed a single distinct red fluorescent plaque. Figure 4C for Δ025L−3d shows that a large number of red fluorescent plaques could be seen in the third generation. Multiple strains of Δ025L−3d virus were collected, and the genome was extracted as a template. PCR showed that the foreign gene had been inserted into the genome, and the CGSIV recombinant virus was successfully constructed. The results in Figure 4C for Δ025L−6d show that the red fluorescent plaques were significantly reduced compared with the previous generations after further separation and purification of the collected venom. The red fluorescent ring was also smaller than in the previous generations. Figure 4C for Δ025L−8/9d (+025L) shows that CGSIV−025L was overexpressed in cells, and the amount of red fluorescence increased after virus infection. These results show that after deletion of the 025L gene, the defective virus could not proliferate normally or the Δ025L−CGSIV mutant could not transform into the wild type. After CGSIV−025L was added to cells, the Δ025L−CGSIV mutant strain used 025L in cells to complete its own replication and continued to proliferate. In conclusion, the overexpression, interference, and deletion mutation experiments indicated that CGSIV−025L was an essential gene for CGSIV.

### 3.5. CGSIV−025L Interacts with CGSIV−062L

**Figure 5:**
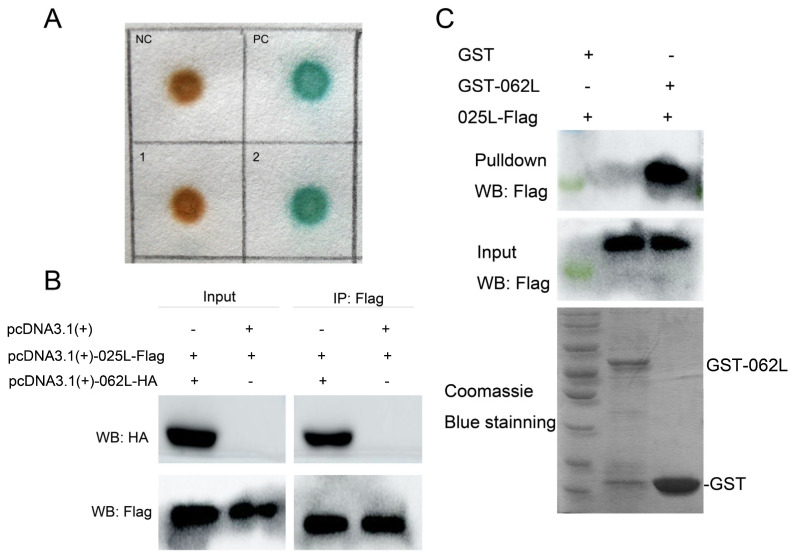
CGSIV−025L interacts with CGSIV−062L. (**A**) Identification of the interaction between CGSIV−025L and CGSIV−062L using yeast two−hybrid screening. (**B**) EPC cells were co−transfected with HA−labeled CGSIV−062L and Flag−labeled CGSIV−025L, and cells were lysed 48 h after transfection for immunoprecipitation assay. CoIP was immunoblotted with anti−Flag antibody and anti−HA antibody. (**C**) GST pulldown was performed with anti−GST antibody, and immunoblotting was performed with anti−Flag antibody.

These results indicate that CGSIV−025L is an essential gene in the virus and can promote virus replication. However, CGSIV−025L has many functions, which may interact with proteins related to viral DNA replication. Helicase is responsible for the opening of DNA duplexes and the synthesis of new strands during DNA replication, playing an essential role in DNA repair and recombination. In order to investigate the viral proteins interacting with CGSIV−025L, we screened the viral proteins interacting with CGSIV−025L by yeast two−hybrid assays. The results of the yeast two−hybrid assays are shown in Figure 5A and indicate that single colonies grew on the SD/2−plate. The positive control group (PC) and pGADT7−062L and pGBKT7−025L co-transfer group showed positive results for β−galactosidase activity detection. However, SD/4− clones did not grow in the negative control group (NC) and pGADT7−067L and pGBKT7−025L co-transmutation group, indicating that CGSIV−025L interacts with CGSIV−062L, but not with CGSIV−067L. CGSIV−062L encodes a helicase. CGSIV−062L belongs to the DEAD−like helicase superfamily. A diverse family of proteins is involved in ATP−dependent RNA or DNA unwinding. This region contains the ATP binding region and the putative Mg^2+^ binding site.

To further verify whether CGSIV−025L interacted with CGSIV−062L in vivo, we co−transfected the pcDNA3.1−025L−Flag and pcDNA3.1−062L−HA plasmids into EPC cells and collected cell proteins 48 h later and performed an IP assay with anti−Flag antibody. The CoIP experiment results shown in Figure 5B indicate that HeLa cell lysates overexpressing both CGSIV−062L−HA and CGSIV−025L−Flag were incubated with anti−Flag M2 Gel, and the CGSIV−062L−HA fusion protein could be detected. The results showed that CGSIV−025L and CGSIV−062L−HA could interact in vivo.

To further study whether CGSIV−025L directly interacts with the CGSIV−062L protein in vitro, the corresponding GST−062L protein and CGSIV−025L−Flag protein were extracted, and the anti−GST antibody was used for a pulldown assay. GST pulldown results are shown in Figure 5C. At the same time, the GST−062L protein, GST protein, and 025L−Flag protein were incubated. After the agarose GST beads were incubated with anti−GST antibody, a Western blot was performed with the Flag antibody, and the 025L−Flag incubated with the GST−062L protein could be examined. However, 025L−Flag incubated with the GST protein was not observed. The results showed that the GST−062L protein could bind to the 025L−Flag protein, but the GST protein could not bind to the 025L−Flag protein.

In conclusion, the results of the GST pulldown and CoIP experiments were consistent with those of the yeast two−hybrid experiments, which further confirmed the interaction between CGSIV−025L and CGSIV−062L. Thus, CGSIV−025L may be involved in viral infection by participating in viral DNA replication and interacting with helicase and other related proteins.

## 4. Discussion

Ranavirus is a serious pathogen that has caused a variety of catastrophic diseases in ectothermic vertebrates [3,38]; however, our understanding of how these viral genes play a role in the replication of viral infections is limited. Ranaviruses are also classified as notifiable viruses by the World Organization for Animal Health (www.oie.int/eng/en_index.htm, accessed on 1 January 2023). At present, only CGSIV MCP [8], Ambystoma tigrinum virus (ATV) 11R, and 53R [31] are essential genes of ranavirus that have been proven experimentally. Therefore, it is very important and critical to understand how the essential genes of ranaviruses affect virus infection and replication. Bioinformatics found that the PCNA gene exists in all sequenced iridoviruses, and the protein encoded by CGSIV−025L also has homology with PCNA [8]. However, in what manner CGSIV−025L affects viral replication remains unclear. In this study, the effects of CGSIV−025L on viral infection and replication were investigated using dual−luciferase reporter assays, transcription assays, drug inhibition assays, and overexpression, knockdown, and deletion recombination techniques, which helped us to further understand the functions of CGSIV−025L and the role of PCNA−like proteins in viral infection and replication.

This study demonstrates for the first time that CGSIV−025L is expressible and that the CGSIV−025L promoter has CGSIV−dependent initiation specificity in initiation transcription. The CGSIV−025L promoter initiates transcription upon CGSIV infection (Figure 1A). This is consistent with the results of Remziye’s study on Chilo Iridescent virus (CIV) [39], which also established that the two late CIV genes, DNApol and MCP promoters, initiate transcription only after virus infection, indicating that iridovirus genes may have their own viral−dependent initiation specificity in initiation transcription. Temporal expression profiling analysis combined with drug inhibition analysis demonstrated that CGSIV−025L was an early gene, and its transcription level was gradually upregulated with viral infection (Figure 1B), indicating that CGSIV−025L also played a role in the late stage of viral infection. Kaposi’s sarcoma−associated herpesvirus (KSHV) ORF59 is also a PCNA−like protein, which is required for viral DNA polymerase localization to the lytic replication origin (oriLyt), and ORF59 interacts with the chromatin complex at the late stage of viral infection to promote viral replication and assembly [40]. Therefore, CGSIV−025L may be involved in virus assembly and translocation in the late stage of virus infection. The drug inhibition experiment established that the transcription phase of CGSIV−025L is an early gene, which is similar to the observation that PCNA is an IE and E phase gene in most ranaviruses (reference), but the difference is that Sun defined the RGV PCNA as an L phase gene in his study on RGV [30]. These results indicate that the time and function of PCNA in different ranaviruses may be diverse.

The plasmid overexpressing CGSIV−025L effectively upregulated CGSIV−025L mRNA levels up to 32−fold in virus−infected EPC cells (Figure 2A). CGSIV−025L overexpression promoted CGSIV replication and the production of infectious progeny virions (Figure 2B). Further studies revealed that CGSIV−025L overexpression promoted CGSIV DNA replication (Figure 2C). The results demonstrated that CGSIV−025L may promote the replication of CGSIV DNA by stimulating the replication of CGSIV DNA. siRNA interference effectively reduced CGSIV−025L mRNA levels to 10% in virus−infected EPC cells (Figure 3A). siRNA knockdown of CGSIV−025L expression attenuated the CGSIV viral titer (Figure 3B) and CGSIV DNA replication (Figure 3C), suggesting that the siRNA knockdown of CGSIV−025L expression may diminish CGSIV replication by attenuating CGSIV DNA replication. Whitley [41] also demonstrated that the MCP and DNA methyltransferase genes related to viral replication are essential for ranavirus replication using RNA interference technology. In their studies on the RNA interference of frog virus 3 (FV3) MCP and 18K, Robert [42] found that the knockdown of MCP led to a significant reduction in viral titers, while the inhibition of 18K synthesis could not prevent the formation of virions. These results indicated that the deletion of variant genes in ranaviruses had contrasting effects on virus replication. Specifically, 18K was not necessary for virus replication, but MCP and PCNA were indispensable. A Δ025L−CGSIV−deficient virus strain was constructed by homologous recombination technology. Experimental results demonstrated that the defective virus (with RFP fluorescence) with deletion of the 025L gene was less present in progeny (Figure 4C), and the defective CGSIV with deletion of the 025L gene could not replicate to produce sustainable viral progeny. This indicated that CGSIV with deletion of the 025L gene could not replicate. However, the replication arrest of Δ025L−CGSIV caused by the 025L gene defect was rescued after 025L replacement (Figure 4C). Therefore, this research proved that CGSIV−025L is an essential gene of CGSIV through overexpression, interference, and deletion mutation experiments. Our results are consistent with the conclusions of Mariah M [31], who constructed the deleted essential gene of ATV by homologous recombination technology. Viruses lacking essential genes cannot replicate to produce progeny virions. Studies have found that the EBV BMRF1 protein can form homomeric proteins to participate in the formation of the viral replication compartment, which is crucial for viral genome replication [43]. The RSIV PCNA protein specifically colocalized with viral nucleic acid in the viral assembly sites in the cytoplasm of infected hosts [30], suggesting that the ranavirus PCNA may also be closely associated with the formation of the viral replication chamber and virion packaging. It is possible that, due to these essential functions, PCNA is an essential gene for ranaviruses.

These studies confirmed that CGSIV−025L is an essential gene of CGSIV and can promote virus replication. However, there are many mechanisms by which 025L promotes viral replication. To illustrate this, Kaposi’s sarcoma−associated herpesvirus (KSHV) ORF59 has been exhibited to interact with chromatin complexes to stimulate viral replication [40]. Helicase is responsible for opening DNA duplexes in the process of DNA replication, participates in the synthesis of nascent strands, and plays an essential role in both DNA repair and recombination [44]. In this study, viral proteins interacting with CGSIV−025L were screened by yeast two−hybrid assays to acquire replication−related protein helicase (CGSIV−062L), and the direct interaction between CGSIV−025L and CGSIV−062L was further confirmed usng GST pulldown and CoIP experiments. Ke found that the DNA replicas of the giant salamander iridescent virus contained viral PCNA [9]. These results indicated that the PCNA protein was localized to the replicator of viral DNA replication in the intracellular space, because 062L has an ATP−dependent RNA or DNA helicase function, and this region contains an ATP−binding region. These results suggested that PCNA could effectively unbind the virus double strand and fix ATP in the process of viral replication by interacting with 062L, thus promoting viral replication. However, the direct interaction between CGSIV−025L and CGSIV−062L established in this study indicates that CGSIV−025L may be involved in viral infection due to its participation in viral DNA replication, as well as its interaction with related proteins. Nevertheless, how the interaction between CGSIV−025L and CGSIV−062L is involved in CGSIV infection requires further investigation. In addition, CGSIV−025L was found to interact with host proteins, namely DNAJA4 and hnRNPLL (reported elsewhere).

## 5. Conclusions

In summary, our studies of the transcription, overexpression, knockdown, and deletion of CGSIV−025L confirmed that CGSIV−025L is an essential gene of CGSIV, and CGSIV−025L can promote viral replication and interact with helicase. This study enriches the evidence that the PCNA protein is involved in viral DNA replication. In addition, the molecular mechanism of the interaction between CGSIV−025L and helicase needs to be further elucidated. This study could provide clues for the further study of the function of CGSIV−025L and lay the foundation for an understanding of the mechanism of CGSIV infection and virus replication.

## Figures and Tables

**Table 1:** Sequence of primers used in this study.

Primer Name	Primer Sequence
025L−F1	CCCAAGCTTATGCTGTGGGAAGCCGTAA
025L−R1	GGAATTCTGCCCTCAAAGAGAGTCACGG
025L−F2	CGGAATTCGCCACCATGCTGTGGGAAGCCGTA
025L−R2	GCGTCGACTTAGCCCTCAAAGAGAGTCACG
025L−F3	GGGGTACCCTCGTAACGACTG
025L−R3	CCCAAGCTTTGACTGGTTTATC
puro−F	CAGTGTGGTGGAATTATGACCGAGTACAAGCCCACG
puro−R	AGTAGCTCCGCTTCCGGCACCGGGCTTGCG
RFP−F	GGAAGCGGAGCTACTAAC
RFP−R	AAACGGGCCCTCTAGTCATCTGTGCCCCAGTTTG
062L−F1	CGGAATTCGCCACCATGGCAAAACTTTTAAGGC
062L−R1	CCCTCGAGCTACCTCTGCGGTCGTCG
062L−F2	CGGAATTCGCCACCATGGCAAAACTTTTAAGGC
062L−R2	CCCTCGAGTTAAGCGTAATCTGGAACATCGTATGGGTACCTCTGCGGTCGTCG
qICP−46−F	GGTCCTTGTTCAGATTCGC
qICP−46−R	AATCAGGGCTCTGGTTATG
qMCP−F	CTGGAGAAGAAGAATGGGAGGGG
qMCP−R	CTTTCGGGCAGCAGTTTTCGGTC
q025L−F	ATCTTGACGAGCCTGGACATCTTT
q025L−R	GTCCTCGGTCTTTACCAGCTACGT
β−actin−F	CACTGTGCCCATCTACGAG
β−actin−R	CCATCTCCTGCTCGAAGTC
q18S−F	ATGGTACTTTAGGCGCCTAC
q18S−R	TATACGCTATTGGAGCTGG

## Data Availability

The CGSIV–025L sequence of CGSIV obtained in the study has been submitted into GenBank under accession number KF512820.1.
